# Comparison of lithium levels between suicide and non-suicide fatalities: cross-sectional study

**DOI:** 10.1192/j.eurpsy.2024.723

**Published:** 2024-08-27

**Authors:** S. Ando, H. Suzuki, T. Matsukawa, S. Usami, H. Muramatsu, K. Yokoyama, Y. Okazaki, A. Nishida

**Affiliations:** ^1^The University of Tokyo, Tokyo; ^2^Jichi Medical University, Tochigi; ^3^ Juntendo University; ^4^Tokyo Medical Examiner’s Office, Tokyo; ^5^Michinoo Hospital, Nagasaki; ^6^Tokyo Metropolitan Institute of Medical Science, Tokyo, Japan

## Abstract

**Introduction:**

Ecological studies have suggested the protective effect of micro-dose lithium in drinking water against suicide, however, the association between body lithium level and suicide is unknown.

**Objectives:**

We aimed to compare body lithium levels between suicide and non-suicide fatalities.

**Methods:**

This cross-sectional study included 12 suicides and 16 non-suicides who were examined or dissected at the Tokyo Medical Examiner’s Office from March 2018 to June 2021. The aqueous humor lithium concentration was measured twice using inductively coupled plasma mass spectrometry. Analysis of covariance (ANCOVA) was used to compare the lithium concentration between suicides and non-suicides. Mixed-effects model was conducted to account for all lithium concentration data.

**Results:**

The aqueous humor lithium concentration did not change after death (*t*(7)=-0.70, SE=0.03, 95% CI=[-0.09, 0.05], P=0.51, Cohen’s *d*=0.01). The aqueous humor lithium concentration was lower in suicides (mean 0.50 μg/L (variance *s^2^* 0.04)) than in non-suicides (mean 0.92 μg/L (*s^2^* 0.07)) (*t*(26)=4.47, SE=0.09, 95% CI=[0.22 to 0.61], P<0.001, Cohen’s *d*=1.71). The ANCOVA showed that death by suicide was significantly associated with lower lithium concentration (*F*(1, 24)=8.57, P=0.007), and the effect size was large (*η_p_^2^*=0.26). The random intercept model showed a significant effect of suicide on aqueous humor lithium concentration (b=-0.261, SE=0.102, 95% CI=[-0.471 to -0.051], t(24)=-2.568, P=0.017).

**Conclusions:**

The results of this study demonstrate that even micro-dose lithium is associated with suicide death. Clinical studies are warranted to examine the effects of micro-dose lithium on suicide prevention.

**Disclosure of Interest:**

None Declared

